# Characterization of Ornithobacterium hominis colonization dynamics and interaction with the nasopharyngeal microbiome in a South African birth cohort

**DOI:** 10.1099/mgen.0.001635

**Published:** 2026-05-18

**Authors:** Celine C. De Allende, Susannah J. Salter, Siobhan E. Brigg, Micaela Boardman, Shantelle Claassen-Weitz, Kilaza S. Mwaikono, Lesley Workman, Heather J. Zar, Mark P. Nicol, Julian Parkhill, Felix S. Dube

**Affiliations:** 1 Department of Molecular and Cell Biology & Institute of Infectious Disease and Molecular Medicine, University of Cape Town, Cape Town, South Africa; 2 Department of Veterinary Medicine, University of Cambridge, Cambridge, UK; 3 Department of Chemistry, University of the Western Cape, Cape Town, South Africa; 4 Department of Pathology, University of Cape Town, Cape Town, South Africa; 5 Department of Science and Laboratory Technology, Dar es Salaam Institute of Technology, Dar es Salaam, Tanzania; 6 Department of Paediatrics and Child Health, Red Cross War Memorial Children’s Hospital, and SAMRC Unit on Child & Adolescent Health, Cape Town, South Africa; 7 Marshall Centre, Division of Infection and Immunity, School of Biomedical Sciences, University of Western Australia, Perth, Australia

**Keywords:** nasopharyngeal microbiome, *Ornithobacterium*, transposon

## Abstract

*Ornithobacterium hominis* is a recently described Gram-negative bacterium that colonizes the human nasopharynx and may be associated with poor upper respiratory tract health. Here, we describe the isolation of *O. hominis* from samples collected from a South African birth cohort, creating the first archive of cultured strains of the species from Africa. Sequenced genomes from this archive reveal that South African *O. hominis* is more similar to Australian strains than those from Southeast Asia and that it may share genes with other members of the microbiome that are relevant for virulence, colonization and antibiotic resistance. Leveraging existing microbiome data from the cohort, *O. hominis* was found to be closely associated with bacterial co-colonizers that are rare in non-carrier individuals, including *Suttonella*, *Rappaport*, *Helcococcus*, *Lwoffella*, *Moraxella* and *Gracilibacteria*. Their collective acquisition has a significant impact on the diversity of nasopharyngeal communities that contain *O. hominis*. Individuals who have not yet acquired *O. hominis* have a higher abundance of *Lwoffella lincolnii* than individuals who never acquire *O. hominis*, suggesting that this could be a precursor state for successful colonization.

**Impact Statement**

First described in 2019, *Ornithobacterium hominis* is an understudied bacterium that may be associated with poor respiratory health in children. The study builds upon existing knowledge of *O. hominis* by describing the first African isolates of the species, its potential to act as a reservoir of virulence and antibiotic resistance genes in the upper respiratory tract and the unique microbiome profile of *O. hominis* carriers. These preliminary data support future work by establishing a starting point for antimicrobial susceptibility testing in a new species and by proposing an association between nasopharyngeal carriage of *O. hominis* and pathobiont species, including *Rappaport israeli* and *Moraxella lacunata*.

## Data Summary

*Ornithobacterium hominis* data have been deposited under project accession PRJEB64694. This comprises genome sequences for isolates SA-OH-C1 (ERR13967269), SA-OH-C2 (ERR13967270), SA-OH-C3 (ERR13967271), SA-OH-C4 (ERR13967272, ERR13967275), SA-OH-C5 (ERR13967273) and SA-OH-C6 (ERR13967274, ERR13967276). Previously published 16S rRNA gene data are deposited under project accessions PRJNA790843 and PRJNA548658. *Helcococcus ekapensis* genome data are deposited under project accession PRJEB85661.

### Software used:

AMRFinderPlus v3.12.8: https://github.com/ncbi/amr

- AssembleBAC-ONT v1.1.1: https://github.com/avantonder/assembleBAC-ONT
- BAKTA v1.8.1: https://bakta.computational.bio/
- blast v2.16.0: https://blast.ncbi.nlm.nih.gov/Blast.cgi
- Comprehensive Antibiotic Resistance Database (CARD) Resistance Gene Identifier (RGI) tool v1.2.1: https://card.mcmaster.ca/analyze/rgi
- Decontam v1.12 (R package): https://github.com/benjjneb/decontam
- Eggnog-mapper v2.0.1: http://eggnog-mapper.embl.de/
- FastANI v1.1.0: https://github.com/ParBLiSS/FastANI
- Flye v2.9.2: https://github.com/fenderglass/Flye
- Guppy v6.5.7: https://community.nanoporetech.com/downloads/guppy/release_notes
- ISEScan v1.7.2.3: https://usegalaxy.eu/root?tool_id=toolshed .g2.bx.psu.edu/repos/iuc/isescan/isescan/1.7.2.3+galaxy1
- Medaka v1.9.1: https://github.com/nanoporetech/medaka
- MEGA11: https://www.megasoftware.net/
- MMseqs2 v17: https://github.com/soedinglab/MMseqs2
- Mothur v1.44.3: https://github.com/mothur/mothur
- NetCoMi v1.2.0 (R package): https://github.com/stefpeschel/NetCoMi
- Panaroo v1.4.3: https://github.com/gtonkinhill/panaroo
- PHASTEST: https://phastest.ca/submissions/new
- Prowler (commit ID c3041ba): https://github.com/ProwlerForNanopore/ProwlerTrimmer
- R v4.4.3: https://www.r-project.org/

### Databases used:

- Comprehensive Antibiotic Resistance Database (CARD): https://card.mcmaster.ca/
- European Nucleotide Archive: https://www.ebi.ac.uk/ena/
- Genome Taxonomy Database (GTDB) release 09-RS220: https://gtdb.ecogenomic.org/
- RefSeq release 228: https://www.ncbi.nlm.nih.gov/refseq/about/prokaryotes/
- SILVA v132: https://www.arb-silva.de/

## Introduction

*Ornithobacterium hominis* is a Gram-negative bacterium that can colonize the human nasopharynx persistently and at high abundance [1]. Genomes from this species were first described from metagenomic data from Thailand in 2019 [2] and from the only cultured isolates thus far, from Australia [3]. *O. hominis* has since been identified in microbiome data from countries across the world, including South Africa [4], and carriage has been reported as associated with poor upper respiratory tract health, such as purulent rhinorrhoea and recurrent otitis media [5], with peak nasopharyngeal abundance observed during or within a week of acute respiratory infection [1].

While lower respiratory tract infections are a major cause of morbidity and mortality in children worldwide, especially in low- and middle-income countries [6], invasive bacterial infection is often preceded by pathogen colonization of the upper respiratory tract such as for *Streptococcus pneumoniae* [7], *Mycoplasma pneumoniae* [8] or *Staphylococcus aureus* [9]. The detection of *Moraxella catarrhalis* in sputum is associated with reduced lung function in children with HIV-associated chronic lung disease (HCLD) [10], but nasopharyngeal density of this species also correlates with HCLD exacerbations [11]. The nasopharynx therefore harbours a diverse community of commensal and pathobiont micro-organisms engaging in complex relationships. Some pathogens in this context have been shown to negatively impact one another’s growth, for example, the inverse correlation of *S. pneumoniae* and *St. aureus* carriage due to pneumococcal H_2_O_2_ production and hydroxyl radical conversion [12,13]. The ‘health associated’ bacterium *Dolosigranulum pigrum* can also inhibit the growth of *St. aureus* and, in combination with *Corynebacterium*, inhibit the growth of pneumococci [14]. Conversely, bacteria may rely on one another for nutrients to support growth, such as *Haemophilus influenzae* benefitting from factor V (NAD) released by the haemolytic activity of *St. aureus* [15]. *O. hominis* possesses potential virulence factors, including *β*-haemolytic activity, similar to that reported in the related avian pathogen *Ornithobacterium rhinotracheale* [16] and a conserved *Pasteurella multocida*-like toxin predicted to target host cell heterotrimeric G-proteins [2]. These factors may facilitate survival of the bacterium in the nasopharynx as well as modifying conditions to the benefit of co-colonizers. Little is known about the organism’s interactions with other members of the microbiome, and, as a persistent colonizer, its potential as a reservoir of antibiotic resistance or virulence factors is a relevant concern.

In order to address these fundamental gaps in our knowledge about *O. hominis*, we aimed to investigate the genetic diversity and colonization dynamics of *O. hominis* in a South African birth cohort and to describe the differential microbiome profiles of children colonized with *O. hominis* compared to uncolonized counterparts.

## Methods

### Sampling and participants

The Drakenstein Child Health Study (DCHS) [17] is a population-based birth cohort in which pregnant mothers in Paarl, South Africa, were enrolled during their second trimester and mother–child pairs have been followed from birth until at least when the children reach adolescence. Nasopharyngeal swabs were collected every 2 weeks from birth through to 1 year of age in an intensive cohort or at well baby visits until 12 months of age and every 6 months thereafter. Archived nasopharyngeal samples were stored at −80 °C. Ethical approval was received from the Human Research Ethics Committee of the University of Cape Town, South Africa (401/2009 and 585/2015). Mothers participating in the parent study provided informed, written consent for enrolment of their infants at the time of delivery and annually.

### 16S rRNA gene data and screening

In order to target culture efforts, data from a prior DCHS microbiome study [4] were used to identify archived samples with a high proportion of *O. hominis*: 6,025 nasopharyngeal specimens and 330 induced sputum samples from 608 infants were examined through 16S rRNA gene sequencing targeting the V4 region, along with 754 technical controls. Detailed library preparation and sequencing methods have been described previously [18].

To enable rapid identification of *O. hominis* in a large set of samples, the amplicon dataset was screened for perfect matches to three sequences unique to this species in the 16S V4 region: [GAGCGTTATCCGGATTCATTGGGTTTAAAGGGTCYGTAGGCGGGCTRATAAGTCAGTGGTGAAATCTCAC], [GAGTGAGTTTGATGTTGCTGGAATGTGTAGTGTAGCGGTG] and [ATGCGAAGGCAGGTAACAAAGACTTAACTG].

### *O. hominis* isolation from DCHS nasopharyngeal samples

Based on 16S rRNA gene abundance data and prior metagenomic data, 36 nasopharyngeal samples with >1% relative abundance of *O. hominis* reads were selected for isolation attempts, giving rise to six isolates. Nasopharyngeal swabs were inoculated onto Columbia agar plates supplemented with 5% sheep’s blood (CBA) and incubated at 30 °C for at least 120 h under microaerophilic conditions. Oxoid^™^ CampyGen^™^ 2.5 L sachets (ThermoFisher Scientific) were used to generate a microaerophilic environment. Presumptive *O. hominis* colonies, as described previously [3], were selected and subcultured on CBA and incubated at 30 °C for at least 48 h. Colonies from primary culture appeared greyish, punctiform and only apparent after 5 days of incubation. Colonies were subcultured until pure cultures were obtained.

Colonies were subjected to PCR targeting the conserved *O. hominis toxA* gene, using a procedure modified from that described previously [2]. Briefly, a single colony was transferred to 200 µl of AVE buffer (Qiagen, Germany) using a sterile loop and heated at 95 °C for 5 min. The lysate was diluted 1:10 in nuclease-free water and PCRs were performed using 1 µl of diluted lysate in a final reaction volume of 25 µl with OneTaq^®^ Quick-Load^®^ 2×Master Mix (New England Biolabs) and OH_TOXIN primers (Inqaba biotech, South Africa) targeting the *toxA* gene: OH_TOXIN-F_ii 5′-GATGTATTGATAGATACTCCCGCCATTACG-3′, OH_TOXIN-R_ii 5′-CTATATTTGGGAAAGGCGCATGAATACC-3′. The TOXIN PCR cycling conditions were as follows: initial denaturation at 94 °C for 30 s; 30 cycles of 94 °C for 30 s; 55 °C for 30 s; 68 °C for 1.20 min; final extension at 68 °C for 5 min; hold: 4–10 °C. PCR products were stored at 4 °C and visualized on a 1.6% agarose gel stained with SYBR^™^ safe DNA gel stain (ThermoFisher Scientific). Cultures positive for the *toxA* gene were banked in brain heart infusion broth+50% glycerol solutions and stored at −80 °C for further analysis.

### Antibiotic susceptibility testing

Antibiotic susceptibility was determined using the disc diffusion method. Frozen stocks were thawed and inoculated onto CBA plates, followed by incubation at 30 °C for 72 h. A loopful of colony material was inoculated into 3 ml (~5 mm depth) of Todd Hewitt broth in a flat-bottomed vial, lined with ~1 ml of tryptic soy agar. Liquid cultures were statically incubated for 48 h at 37 °C; thereafter, cultures were resuspended and assessed visually for turbidity. A 1:1 dilution of the culture was made in Todd Hewitt broth and further statically incubated for 3–4 h or until an increase in turbidity was apparent using McFarland standards; this allowed the *O. hominis* growth to recover. A volume of 100 µl of the diluted broth was inoculated onto CBA plates using the spread plate method. Three replicates were included for each strain. Antibiotic discs (Oxoid, ThermoFisher Scientific) of ampicillin (10 µg), amoxicillin (25 µg), vancomycin (5 µg), penicillin G (1 U), azithromycin (15 µg), clindamycin (2 µg), tetracycline (30 µg), trimethoprim-sulfamethoxazole (co-trimoxazole) (25 µg) and polymyxin B (300 U) were placed onto the agar surface using sterile forceps. Plates were incubated for 48 h at 30 °C under microaerobic conditions. Growth was evaluated and zone sizes were measured using callipers.

Nitrocefin discs (Remel, ThermoFisher Scientific) were used according to the manufacturer’s instructions. In brief, discs were heavily inoculated with bacterial growth from solid media and incubated for 5 min in air at room temperature. A colour change from white to pink indicated a positive result for *β*-lactamase production, while no colour change indicated a negative result. Isolates were tested in triplicate.

### DNA extraction and sequencing

Confirmed *O. hominis* isolates were cultured on CBA and incubated at 30 °C for up to 120 h for DNA extraction. Genomic DNA was extracted from pure bacterial cultures using the Wizard HMW DNA extraction kit (Promega, USA). The protocol was performed according to the manufacturer’s instructions with some modifications. Briefly, colonies were scraped from plates and pelleted in PBS by centrifugation at 16,000 ***g*** for 2 min. The supernatant was discarded and the cell pellet was resuspended in PBS. Resuspensions were incubated at 85 °C for 5 min. Briefly, 500 µl lysis buffer was added along with 6 µl RNase A and the mixture was incubated at 37 °C for 30 min. Briefly, 20 µl of Proteinase K was added and the samples were incubated at 56 °C for 15 min. Protein was precipitated using protein precipitation solution. Thereafter, the samples were centrifuged at 16,000 ***g*** for 10 min at room temperature. The supernatant was carefully transferred into room temperature isopropanol for DNA precipitation. The samples were centrifuged at 16,000 ***g*** for 2 min to pellet the DNA. The DNA pellet was washed with 70% ethanol and the samples were centrifuged at 16,000 ***g*** for 2 min before allowing the DNA pellet to air dry at room temperature. The DNA was left to resuspend in 50 µl of DNA resuspension buffer at room temperature. DNA was stored at 4 °C until ready for further analysis.

The sequencing library was prepared with the native barcoding kit 24 v12 (SQK-NBD112.24; Oxford Nanopore Technologies) according to the manufacturer’s guidelines. Briefly, DNA was FFPE repaired and end-prepped/dA-tailed, then a unique dT-tailed barcode adapter was ligated on the dA-tailed template. Barcoded samples were pooled and sequencing adapters were ligated. The library was sequenced on an Oxford Nanopore MinION platform using a flow cell R10.4 (FLO-MIN114; Oxford Nanopore Technologies).

### Genome analysis

Base calling and barcode removal were undertaken using the SUP model in Guppy v6.5.7 (GPU). Reads were trimmed using Prowler [19] to a Q20 average in a 1,000-nucleotide window, discarding reads of length <1 kb. Preliminary assemblies were generated with Flye v2.9.2 [20] using default settings. Genomes were circularized and rotated to the origin of replication as described previously [21]. The genome was polished with Medaka v1.9.1 using trimmed reads. Finally, the genome was annotated with Bakta v1.8.1 [22]. Due to poor performance of isolate SA-OH-C4 specifically, an alternative pipeline was used in this case: AssembleBAC-ONT, developed by Andries van Tonder (https://github.com/avantonder/assembleBAC-ONT). Briefly, Artic guppyplex aggregated pre-demultiplexed reads from Guppy. Long reads were filtered using Filtlong, discarding reads of <5 kb. As with the other genomes, AssembleBAC-ONT assembled reads with Flye, polished with trimmed reads using Medaka and annotated with Bakta.

**Fig. 1.**
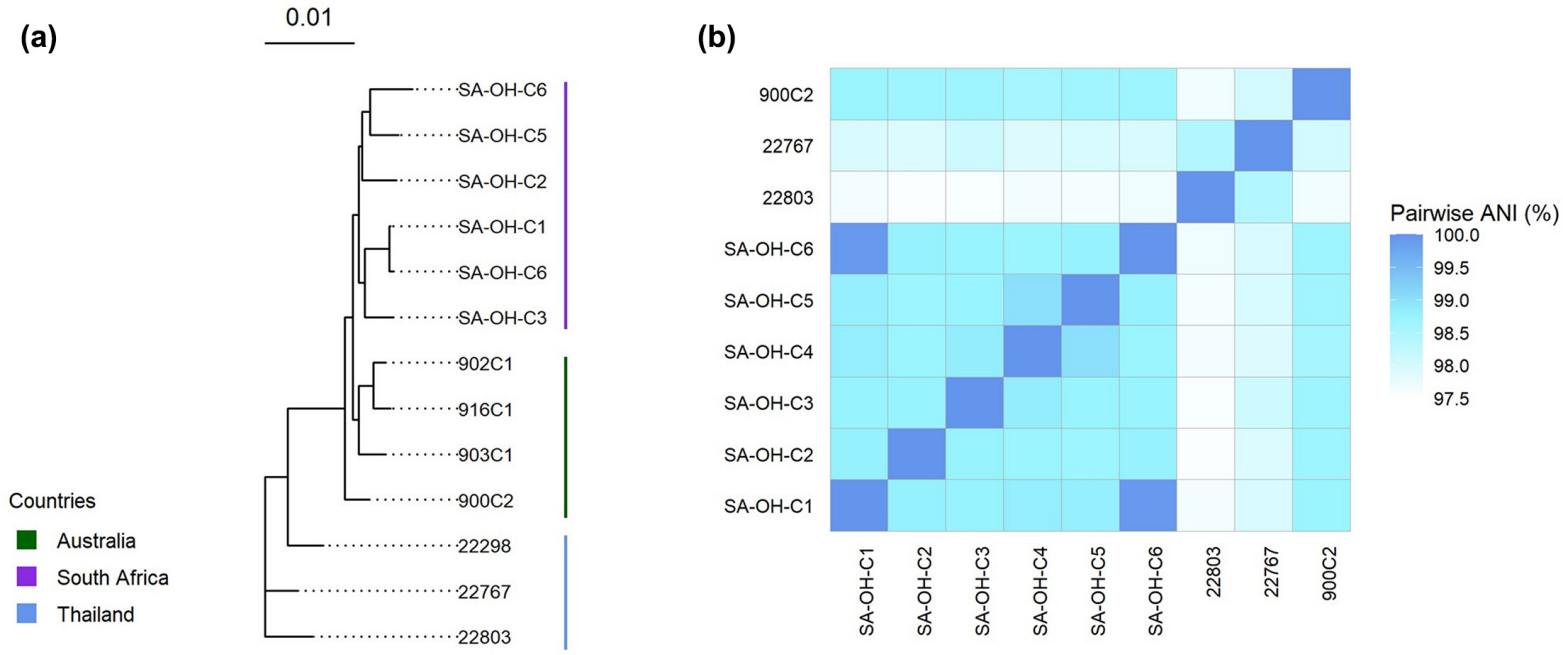
(a) Maximum likelihood phylogenetic tree of core genes from South African (purple), Thai (blue) and Australian (green) *O. hominis* genomes. (**b**) Heatmap of pairwise ANI.

Antimicrobial resistance genes were identified using NCBI’s AMRFinderPlus tool v3.12.8 [23] and the Comprehensive Antibiotic Resistance Database (CARD) resistance gene identifier (RGI) tool v1.2.1 [24]. Pangenome analysis was performed using Panaroo [25]. The pangenome analysis included annotated assemblies of South African, Thai and Australian *O. hominis* isolates publicly available under accessions PRJEB64694, PRJEB25749 [2] and PRJNA510696 [3], respectively. Panaroo was used with default settings in the strictest mode and generated a core genome alignment (all genes present in 98% of isolates) using the clustalW multiple sequence aligner. The core genome alignment tree (Fig. 1a) was calculated in MEGA11 and visualized using ggtree in R v.4.4.2. Evolutionary history was inferred using the maximum likelihood method and Tamura–Nei model. The bootstrap consensus tree inferred from ten replicates was taken to represent the evolutionary history of the taxa analysed. Branches corresponding to partitions reproduced in less than 50% bootstrap replicates are collapsed. The percentage of replicate trees in which the associated taxa clustered together in the bootstrap test are shown next to the branches. Pairwise whole-genome average nucleotide identity (ANI) was calculated using FastANI v1.1.0 [26] with default parameters. Functional prediction of core genes was performed using eggNOG-mapper [27] with bacteriophage detection using PHASTEST [28] and insertion sequence (IS) element prediction with ISEScan v1.7.2.3 [29]. Comparison of amino acid sequences was undertaken with MMseqs2 v17 [30] using default settings with a custom database comprising all 99,432 RefSeq genomes [31] that belonged to a genus present in at least 1% abundance in at least one sample in the reported microbiome analysis, plus the top 20 genera reported for the full cohort [4]. Further details are reported in the Supplemental Methods.

### Microbiome analysis

Using the 16S rRNA dataset described above, samples were selected from 23 infants with the highest predicted *O. hominis* abundance (*O. hominis* carrier group, timepoint 2). The closest preceding sample that had no *O. hominis* reads was selected as a pre-colonization comparator (*O. hominis* carrier group, timepoint 1). Non-carriers of *O. hominis* were identified in the dataset based on an absence of *O. hominis* reads from birth to 30 months of age, and 23 of these infants were randomly assigned as paired controls. Age-matched samples were selected (non-carrier group, timepoints 1 and 2). One infant had *O. hominis* in all their preceding samples, so timepoint 1 was omitted for this and the paired control. The negative controls associated with these sequencing runs were included in the analysis for decontamination purposes (see below). The selection criteria and metadata of the carrier and non-carrier groups are described in the Supplemental Methods.

Data from these 90 samples and 14 negative controls were cleaned using Mothur v1.44.3 [32]. In brief, contiguated read pairs were removed if they had any ambiguous bases, homopolymers of length >8 or a total length >260 bp, and following alignment they were trimmed to the expected V4 region of the 16S rRNA gene. Chimaeras were identified using the chimera.vsearch function with dereplication and removed from downstream analysis (in total 7.9% of reads were removed). Finally, reads were classified against the SILVA database v132 [33] and those identified as Chloroplast, Mitochondria, Archaea, Eukaryota or of unknown Kingdom were discarded, as well as actual sequence variants (ASVs) comprising only one read (thus a further 2.9% of reads that passed chimaera detection were removed). The ASV matrix from Mothur was converted to a phyloseq object in R v4.2.1 [34] in order to run Decontam v1.12 [35] using the prevalence method with a threshold of 0.5. Further details of decontamination are described in the Supplemental Methods.

Alpha diversity metrics (coverage, observed ASVs, Inverse Simpson index) were calculated in Mothur with rarefaction to the lowest sample depth, 10,084. Paired t-tests in R were used to compare ASV richness between groups. Due to non-normal distribution, diversity scores were compared with the Mann–Whitney U test.

ASV network analysis was undertaken for well-represented taxa, i.e. all 60 ASVs with a relative abundance of >1% in at least one sample and excluding singletons. Network construction and visualization used NetCoMi v1.2.0 [36] with the SparCC association measure for a fully connected (dense) network, identifying hub nodes by the eigenvector method. Edges were filtered by weight <0.25. ASVs were classified using the RefSeq Targeted Loci database.

## Results

### Identification and recovery of *O. hominis* from nasopharyngeal samples

To aid recovery of *O. hominis* from the DCHS sample archive, microbiome data from the study site were examined. *O. hominis* reads were detected in 629/6,025 nasopharyngeal samples from 204/605 children in the dataset (10.4% nasopharyngeal samples or 33.7% of participants) as well as in 14/330 sputum samples from 12/239 infants (4.2% of sputum samples or 5% of participants). In six of these sputum samples (from five infants), *O. hominis* was also detected in the nasopharynx on the same date or immediately preceding it.

Archived nasopharyngeal samples were selected for isolation attempts based on the following criteria: (i) longitudinal carriage of *O. hominis* (presence of *O. hominis* reads in more than one nasopharyngeal sample from the same child), (ii) availability of child age data and (iii) expected *O. hominis* abundance greater than 1%. In total, 34 nasopharyngeal samples fulfilled these criteria and were targeted for isolation; however, due to slow growth, small colony size and low abundance in the samples, *O. hominis* was successfully isolated from only four of these samples. Two additional isolates (SA-OH-C1 and SA-OH-C5) were cultivated from the DCHS sample archive, but these candidates were not present in the 16S dataset and instead were identified using data from a prior metagenomic study (PRJNA727021, manuscript in preparation).

In summary, six *O. hominis* isolates were recovered and sequenced from samples collected between 2013 and 2016. The age of the children at the time of sampling ranged from 238 days (7.8 months) to 761 days (>2 years of age), and the relative abundance of *O. hominis* in the nasopharyngeal samples was between 1 and 6%. All isolates were obtained from swabs collected during routine study sampling, except for two infants, C1 and C5, who were sampled during acute respiratory illness (Table 1).

Colonies typical of *O. hominis* morphology as described by Lawrence *et al.* were observed; colonies appeared greyish, glistening, convex and circular with smooth edges [3]. Isolate SA-OH-C6 exhibited a consistently mucoid colony morphology. A subculture of a single colony could yield a variety of colony sizes, ranging from punctiform (pinprick-sized, less than 1 mm in diameter) to larger colonies up to 3 mm in size. Uniformly sized colonies of ~1 mm in diameter were also apparent. Notably, punctiform colonies from mixed morphology plates typically become apparent after prolonged incubation and increased in frequency with extended incubation periods. *O. hominis* colonies also exhibited *β*-haemolysis on CBA plates after extended incubation (>96 h). This haemolysis was most pronounced under the primary streak. Haemolytic activity was not observed for the smaller, punctiform colonies. Growth and appearance of colonies became more luxuriant following passage in shallow Todd Hewitt broth.

**Table 1.** Sample information for *O. hominis* isolates

Strain	Date of sampling (year)	Age of infant (days)	Illness/Routine
SA-OH-C1	2014	493	Illness
SA-OH-C2	2013	239	Routine
SA-OH-C3	2014	336	Routine
SA-OH-C4	2014	238	Routine
SA-OH-C5	2015	587	Illness
SA-OH-C6	2016	761	Routine

### Characteristics of South African *O. hominis* genomes

As summarized in Table 2, all genomes were ~2 Mb in size with sequencing coverage ranging from 17× to 55×. Average G+C content was 35.7 mol%, and there were ~2,000 genes in each genome; SA-OH-C2 appears to have more genes than the other genomes, an artificial inflation caused by frameshift sequencing artefacts typical of low-depth ONT sequence data. However, this did not affect downstream analysis due to the successful recovery of fragmented genes by Panaroo. Single, circular chromosomes were generated for all genomes except for SA-OH-C4 which assembled into six contigs. Following analysis with PHASTEST, one of these contigs was found to contain a 33 kb bacteriophage, while another was identified as a putative plasmid based on the assembly statistics (4.5 kb length, circular and 20-fold higher coverage than the bacterial chromosome) and by gene content (including a replication initiation protein, MobV-family relaxase and two VapD-like endoribonucleases).

**Table 2.** *O. hominis* genome characteristics

Genome	Accession no.	Total length (Mbp)	No. of contigs	Complete	Mean coverage (×)	G+C content (mol%)	Total genes (CDS)	Plasmids
SA-OH-C1	ERS21330455	2.05	1	Yes	55	35.75	1,991 (1,934)	0
SA-OH-C2	ERS21330459	2.05	1	Yes	23	35.69	2,183 (2,123)	0
SA-OH-C3	ERS21330456	2.01	1	Yes	51	35.69	1,900 (1,840)	0
SA-OH-C4	ERS21330457	1.99	6	Contigs	50	35.71	1,965 (1,908)	1
SA-OH-C5	ERS21330460	2.05	1	Yes	17	35.49	2,045 (1,987)	0
SA-OH-C6	ERS21330458	2.05	1	Yes	46	35.73	2,089 (2,030)	0

The core genome tree (Fig. 1a) illustrates the relationship between the genomes from South African and published Australian isolates [3] as well as genomes from the Mae La cohort in Thailand [2]. The Australian and South African genomes appear to be more closely related, while the genomes from Thailand are more distantly related. Pairwise ANIs were >97% between the genomes (Fig. 1b, Table S1, available in the online Supplementary Material), confirming that they are members of the same species.

**Fig. 2.**
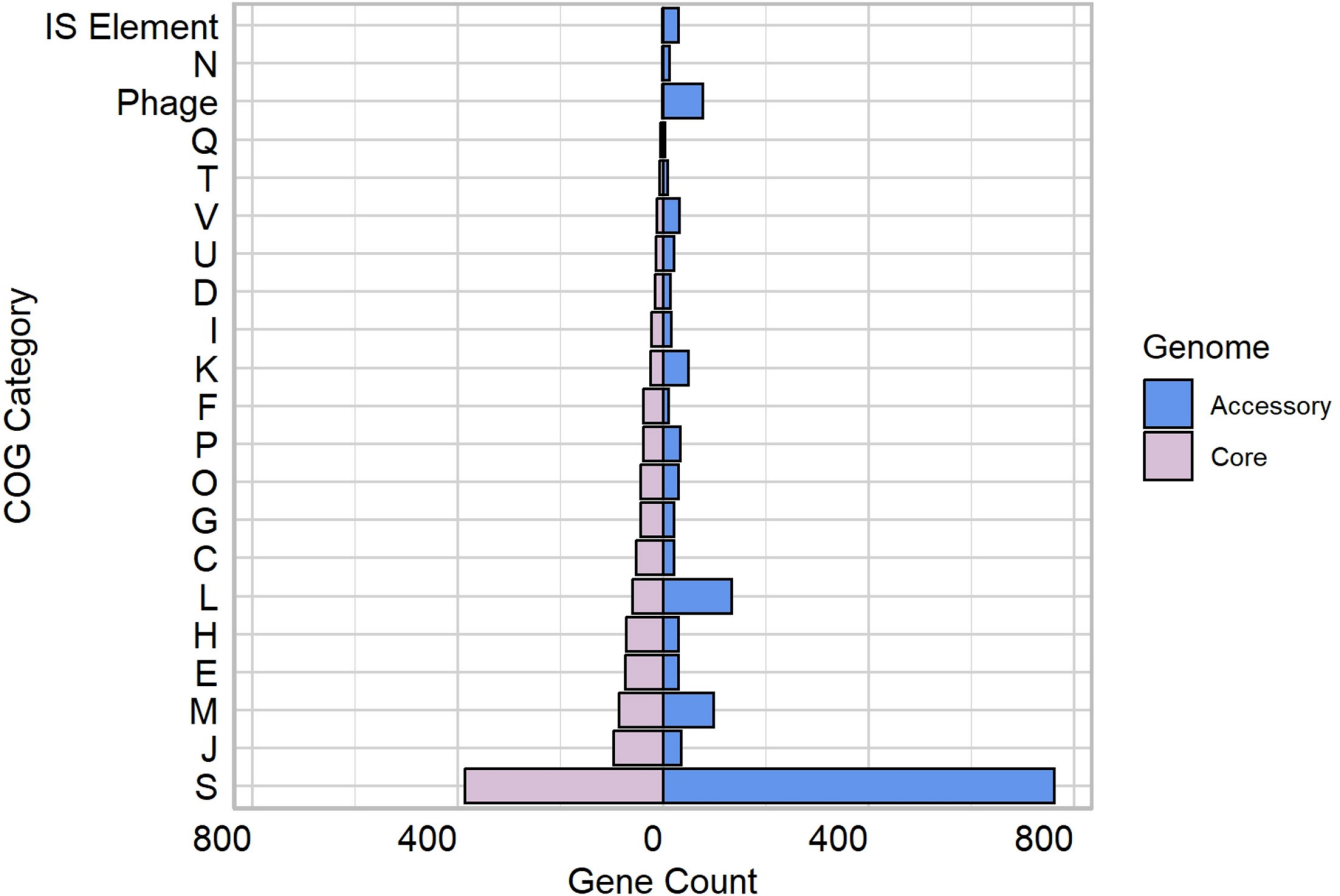
Summary of accessory genome COG groups. C: Energy production and conversion; D: Cell cycle control, cell division, chromosome partitioning; E: Amino acid transport and metabolism; F: Nucleotide transport and metabolism; G: Carbohydrate transport and metabolism; H: Coenzyme transport and metabolism; I: Lipid transport and metabolism; J: Translation, ribosomal structure and biogenesis; K: Transcription; L: Replication, recombination and repair; M: Cell wall/membrane/envelope biogenesis; N: Cell motility; O: Post-translational modification, protein turnover, chaperone functions; P: Inorganic ion transport and metabolism; Q: Secondary metabolites biosynthesis, transport and catabolism; T: Signal transduction; U: Intracellular trafficking, secretion and vesicular transport; S: Function unknown/unassigned; V: Defence mechanisms; and X: Mobilome.

The functions of the accessory and core genes of *O. hominis* were investigated using EggNOG-mapper with supplementary assignment of bacteriophage and IS elements using PHASTEST and ISEScan. Clusters of orthologous groups (COGs) were assigned genes where possible, and the distribution of gene counts across the COG categories is presented in Fig. 2. In total, ~800 COGs were identified for the accessory genome and 900 COGs for the core genome, although a large proportion of the accessory genes (215/800, 26.87%) and core genes (179/900, 19.89%) could not be functionally annotated. The accessory genome annotations were dominated by groups associated with replication and repair, cell wall biogenesis and bacteriophage. The first and second categories are somewhat inflated, as ‘replication and repair’ includes genes from mobile elements such as integrase/recombinases and nucleases, and ‘cell wall biogenesis’ includes 26 orphan tips (pseudogenes) associated with a large RHS (rearrangement hotspot) protein gene. In contrast, genes linked to secondary metabolite biosynthesis and catabolism, signal transduction, nucleotide metabolism and cell motility were poorly represented in the accessory genome.

In the core genome, most genes were associated with translation and ribosome function, cell membrane biogenesis and metabolism of amino acids and coenzymes. Like the accessory genome, core genes related to cell motility and signal transduction were the least represented among the categorized genes in *O. hominis*. No genes were assigned to Z (cytoskeleton), Y (nuclear structure), X (mobilome), W (extracellular structures), R (general function prediction) and A (RNA processing and modification) COG categories.

All six *O. hominis* genomes include novel prophages. SA-OH-C2 encodes three prophage regions, while SA-OH-C1, -C3, -C4 and -C6 have two and SA-OH-C5 has one. Where multiple phages are present, they are dissimilar to one another, except for SA-OH-C4, which includes one intact bacteriophage chromosome and at least one truncated copy of the same prophage inserted into the host chromosome. The variable presence of this prophage may be responsible for the assembled genome’s fragmentation into contigs.

### Antimicrobial resistance potential in *O. hominis*

The AMR Finder and CARD RGI tools were used to screen for AMR genes in the genomes and isolates were evaluated for *β*-lactamase production and susceptibility to commonly received antibiotics in the cohort: amoxicillin, ampicillin, penicillin G, azithromycin, clindamycin, tetracycline and trimethoprim-sulfamethoxazole, as well as polymyxin B and vancomycin (Table 3).

**Table 3.** Antibiotic resistance potential screening of South African *O. hominis* isolates, disc diffusion method

	Strains			
	SA-OH-C2	SA-OH-C4	SA-OH-C5	SA-OH-C6
**Antibiotic**	Zone diameter±sd (mm)			
**Polymyxin B (300 U**)	8.33±0.57	8.66±0.57	7.67±0.57	7.33±1.15
**Vancomycin (5 µg**)	14±1.73	16.3±2.08	13.3±2.08	14.6±2.08
**Amoxicillin (25 µg**)	49.5±3.53	43±2.82	44±2.64	30±2.64
**Ampicillin (10 µg**)	45.5±0.71	53.5±0.71	46±1.73	34.7±2.5
**Penicillin G (1 U**)	50.1±5.37	48.29±1.28	46.35±1.68	11.33±3.01
**Azithromycin (15 µg**)	28.32±0.94	37.58±0.73	34.76±1.31	27.15±0.25
**Clindamycin (2 µg**)	38.42±0.68	45.19±0.99	44.59±1.36	38.5±1.41
**Tetracycline (30 µg**)	40.23±3.56	46.23±1.44	41.12±1.94	40.14±1.20
***β*-Lactamase production (positive/negative**)	Negative	Negative	Negative	Positive
**AMR genes**	*vanT*, *vanY*	*vanT*, *vanY*	*vanT*, *vanY*	*vanT*, *vanY*, *cfxA*
**AMR gene family**	Glycopeptide resistance gene cluster	Glycopeptide resistance gene cluster	Glycopeptide resistance gene cluster	Glycopeptide resistance gene cluster CfxA family broad-spectrum class a *β*-lactamase

An incomplete set of genes associated with vancomycin resistance (v*anT* and *vanY*) was identified in all South African strains. Two strains, SA-OH-C1 and SA-OH-C6, carry the c*fxA* gene, which encodes a class A2 *β*-lactamase that may confer broad activity against *β*-lactam antibiotics. Strains SA-OH-C1 and SA-OH-C3 were not subjected to antibiotic susceptibility testing due to issues with sample viability and contamination; all other isolates were tested in triplicate. Zone diameters are reported in Table 3. Vancomycin inhibition zones were similar across the isolates, ranging from 13.3 to 16 mm. Although European Committee on Antimicrobial Susceptibility Testing breakpoints are not established for amoxicillin and ampicillin in this species, and thus a sensitive/resistant status cannot be inferred, the *β*-lactamase-positive isolate exhibited a reduced inhibition zone for penicillin G compared to the other isolates (11.33±3.01 mm compared to a mean of 48.25 mm). This strain also yielded a modest reduction in zone diameter for ampicillin and amoxicillin antibiotics, respectively.

No inhibition was observed for any isolate exposed to 25 µg co-trimoxazole, suggesting that these isolates possess a resistance mechanism that is not identified by AMR Finder or CARD.

### Exchange of virulence determinants: lipopolysaccharide

**Fig. 3.**
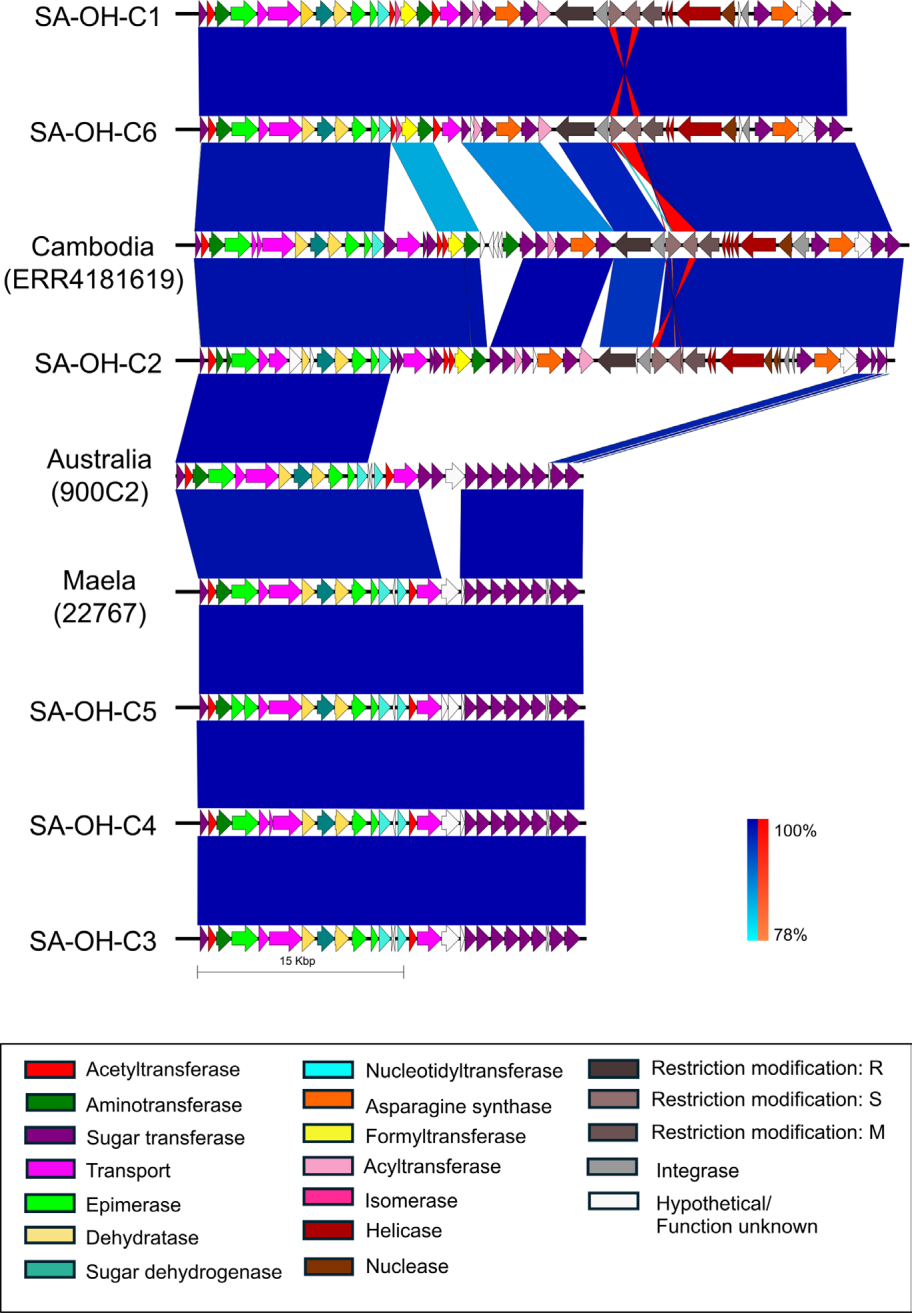
Alignment of LPS biosynthesis clusters in the South African genomes and *O. hominis* from Cambodia, Australia and Thailand. Coding sequence features are marked by arrows and genes were annotated in Bakta. Vertical blocks between sequences indicate regions of shared similarity (dark blue for matches in the same direction or bright orange for inverted matches). Image generated using EasyFig.

The lipopolysaccharide (LPS) is essential for the survival of Gram-negative bacteria in the host environment and is critical for host–microbial interactions; thus, the structure and composition of the LPS biosynthesis locus in the South African genomes were analysed and compared to available LPS clusters from public *O. hominis* data. The alignment (Fig. 3) of the LPS locus illustrates its composition and organization. As described previously [3], the initial 14 kb and two terminal sugar transferase genes are highly conserved across the LPS types, offering a conserved site for variation through homologous recombination. SA-OH-C3, -C4 and -C5 possess an LPS cluster identical to that of the Thai genome OH-22767 (accession no. GCF_900538225.1), demonstrating that the LPS is decoupled from the structure of the core genome tree (Fig. 1a). The LPS clusters from SA-OH-C1, -C2 and -C6, although similar to the Cambodian LPS type (derived from accession no. ERR4181619), exhibit differences in the central region of the cluster. SA-OH-C1 and -C6 include a novel transport gene, possibly a novel flippase, as well as distinct glycosyltransferases and a unique acyltransferase. Compared to SA-OH-C1 and -C6, SA-OH-C2 is more similar to the Cambodian LPS type with shared transport genes; however, -C2 has an additional acyltransferase and lacks the aminotransferase found in that genome.

In South African isolates SA-OH-C1, -C2 and -C6, the LPS gene cluster also encompasses a 15 kb putative non-autonomous transposon. Flanked by two tyrosine type integrase/recombinase genes, the transposon contains a YhcG nuclease gene, two helicase genes and type I restriction enzyme subunits SMR. Although the complete element has not been observed outside of *O. hominis*, the individual genes have a high amino acid identity (>80%) to others within the family, primarily from *Chryseobacterium* spp. The exception is the YhcG nuclease and one helicase, which share 99 and 96% amino acid identity, respectively, with genes from a mobile element in *Sphingobacterium mizutaii* strain SM2 (acc. no. NZ_JACLGQ010000002), a clinical isolate from China. The species *Sp. mizutaii* was originally classified as *Flavobacterium*.

### The nasopharyngeal microbiome of *O. hominis* carriers differs from non-carriers

**Fig. 4.**
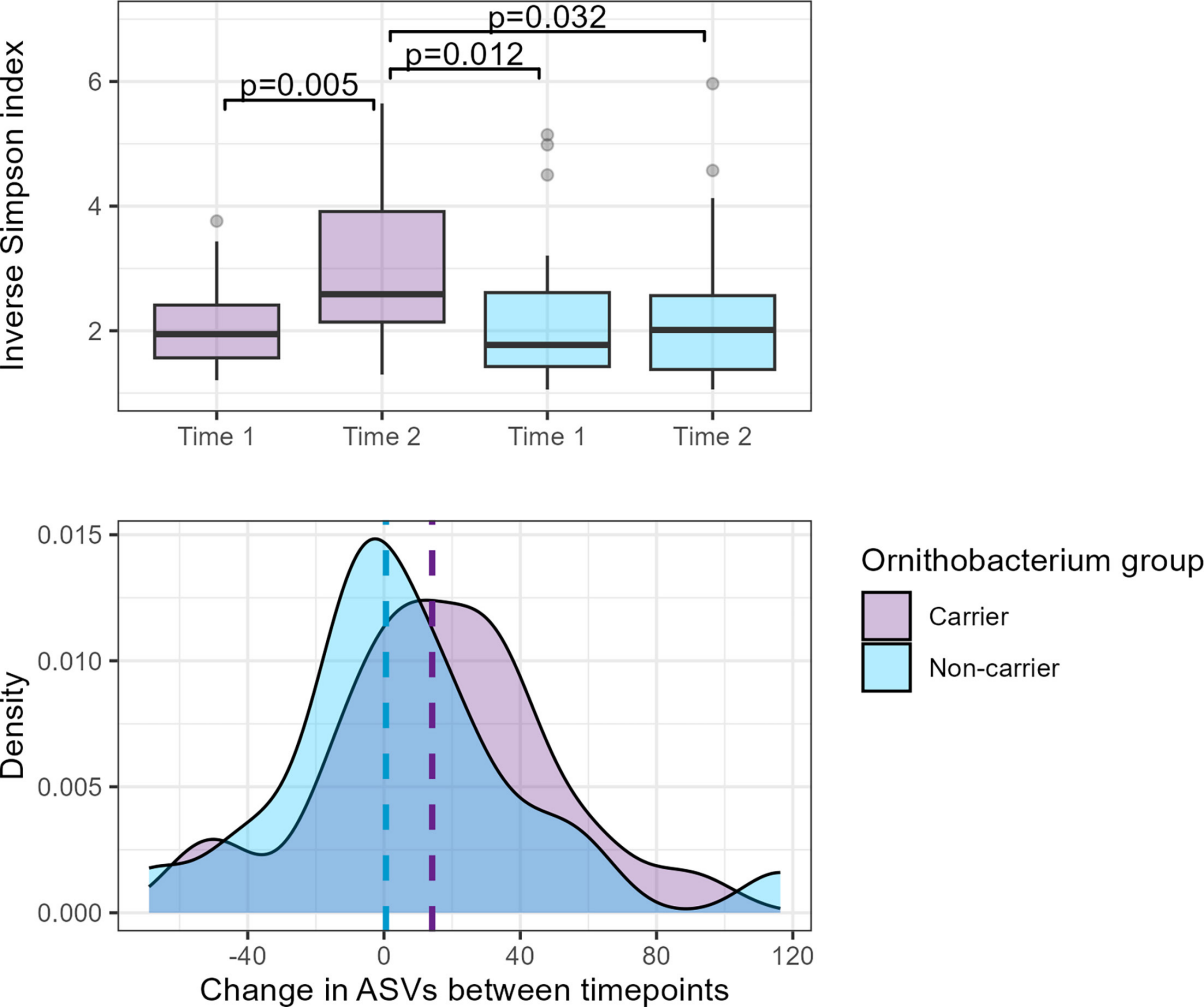
Boxplot (top) of Inverse Simpson diversity scores for *O. hominis* carriers (purple) and non-carriers (blue). The *O. hominis* carrier group at timepoint 2 is significantly more diverse than at the earlier timepoint and in comparison to non-carriers. Distribution plot (bottom) of the change in community richness per infant between timepoints, with the group median indicated by dashed line (*O. hominis* carriers+14.1 ASVs, non-carriers+0.6 ASVs).

Using the screened 16S rRNA gene data as a starting point, matched samples from 23 *O. hominis* carriers with >1% relative abundance (sampled pre- and post-colonization) and 23 non-carriers were examined. To overcome the confounding influence of age on microbiome composition, samples from *O. hominis* carriers and non-carriers were well matched, with a median difference in age of 2 days (maximum difference 37 days) (Supplemental Methods). Alpha diversity measures show increased diversity (Inverse Simpson index) between timepoints 1 and 2 for the *O. hominis* carrier group (*P*=0.005) with no significant change in the non-carrier group; this change is not solely due to the acquisition of *O. hominis*, as the increase in community diversity remains significant when *O. hominis* ASVs are removed from the dataset (*P*=0.022). Furthermore, the diversity scores of samples with *O. hominis* are higher than age-matched samples from non-carriers (*P*=0.032) (Fig. 4). The median increase in community richness (observed ASVs) over time for *O. hominis* carriers was 14.1 ASVs, but the increase was not significant.

**Fig. 5.**
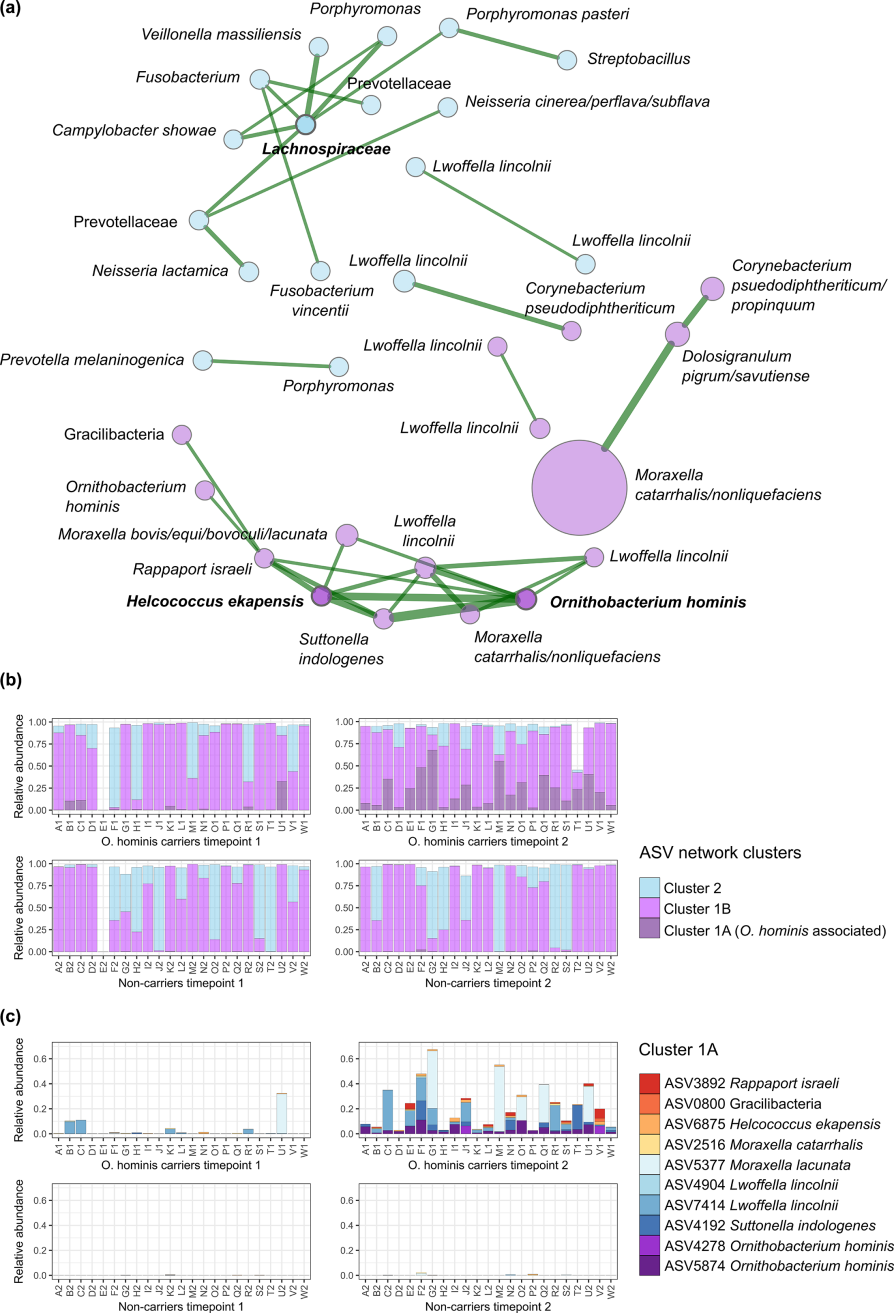
(a) SparCC network diagram illustrating connected ASVs within cluster 1 (purple nodes) and cluster 2 (blue nodes), and all positive associations with weight >0.25 (green edges). Three hub nodes were identified (in bold): *O. hominis, Helcococcus* sp. and an unclassified *Lachnospiraceae*. Node sizes are scaled by normalized counts. (**b**) Relative abundance of the ASV clusters at two timepoints in *O. hominis* carriers (**A1–W1**) and non-carriers (**A2–W2**), with Cluster 1 divided into the *O. hominis-*associated subcluster 1A and non-associated ASVs (1B). (**c**) Composition of subcluster 1A.

A correlation network was calculated using 60 key ASVs, encompassing 96.4% of total reads. As illustrated in Fig. 5(a), the ASVs were categorized into two clusters: Cluster 1 (in purple) that includes a well-connected subcluster with two hub nodes, *O. hominis* and *Helcococcus*, and Cluster 2 (in blue) with one hub node, an unclassified *Lachnospiraceae*.

Subcluster 1A comprises ASVs identified as *O. hominis*, *Rappaport israeli*, unclassified *Gracilibacteria*, *Helcococcus* sp., *M. catarrhalis/nonliquefaciens*, *Moraxella lacunata/bovis/equi/bovoculi*, *Lwoffella lincolnii* and *Suttonella indologenes*. The remainder of Cluster 1 comprises ASVs that are not significantly correlated with *Ornithobacterium*, including *Corynebacterium propinquum/pseudodiphtheriticum*, *D. pigrum/savutiense* and a highly abundant *M. catarrhalis/nonliquefaciens* ASV. Cluster 2 includes several oral-associated taxa, including *Fusobacterium*, *Porphyromonas* and *Veillonella* that may reflect saliva intrusion into the nasopharyngeal sampling site.

Visualizing the clusters present in the *O. hominis* carriers and non-carriers at the pre- and post-colonization timepoints (Fig. 5b, c) illustrates that some *Ornithobacterium*-associated taxa are present before the acquisition of *O. hominis* and that they are less common in the non-carrier group overall. The most abundant of these are the *L. lincolnii*-like ASVs (Fig. 5c).

Isolates SA-OH-C2, SA-OH-C3 and SA-OH-C6 were recovered from infants O1, Q1 and J1, respectively (Fig. 5b, c). Isolates SA-OH-C1, -C4 and -C5 were obtained from infants not included in the paired carrier/non-carrier analysis. During attempts to isolate *O. hominis* from primary samples, bacterial co-colonizers were also recovered at 30 °C under microaerobic conditions. One isolate of *Helcococcus*, SA-HE-C1, was sequenced, yielding a single circular chromosome of 1.4 Mbp and 28.36 mol% G+C content (Table S2).

### *O. hominis* may acquire adaptive genes from the microbial community

Although the number of isolates recovered from the cohort was modest, we sought to identify preliminary evidence for *O. hominis* acquiring adaptive genes from the microbial environment. The *O. hominis* genomes were compared to a custom database of 99,432 reference genomes, including every available species from a genus with sample abundance >1% in the microbiome analysis and the 20 most abundant genera in the full cohort to identify genes that may be shared with other nasopharyngeal species. Five blocks of genes were identified with gene similarity >95% to other genera (Table 4). Of particular interest are the blocks containing genes with amino acid identity >99% that may relate to antibiotic resistance (transposon Tn4555 and an extended spectrum *β*-lactamase gene), adhesion [clostripain and *Fibrobacter succinogenes* domain (FSD) proteins] and toxin production (RHS toxin tip and a putative immunity protein), discussed below.

**Table 4.** All consecutive gene blocks detected in *O. hominis* isolates and in a search database of 99,432 genomes, with amino acid identity >95% over >50% length Accession numbers of top hits are provided or a representative in cases of multiple equally scored hits.

Consecutive gene blocks identified in *O. hominis* and other species	*O. hominis* genomes	Top hit accession no., species, amino acid identity
Transposon Tn4555: TnpA, integrase, TnpC, excisionase, AAA family ATPase, DUF6371 domain-containing protein, hypothetical protein, CfxA family broad-spectrum class A beta-lactamase	SA-OH-C1, -C6	WP_004292844.1 (multispecies 100%), WP_004338318.1 (multispecies 100%), WP_004339687.1 (multispecies 100%), WP_004338328.1 (multispecies 100%), WP_004348225.1 (multispecies 100%), WP_004292742.1 (multispecies 100%), WP_004292734.1 (multispecies 100%), WP_004339683.1 (multispecies 100%)
Clostripain-related cysteine peptidase, preprotein, FSD-containing protein	SA-OH-C1, -C6	WP_172642744.1 (*Prevotella* sp. HJM029 99.5%), WP_106070077.1 (*P. heparinolytica* 99.7%), WP_187545083.1 (*Alloprevotella* sp. Lung230 98.3%)
RHS protein toxin tip, putative immunity protein	SA-OH-C1, -C2, -C3, -C4, -C5, -C6	WP_119059914.1 (*B. cardium* 100%), WP_221411188.1 (*B. cardium* 99.3%)
Helix-turn-helix domain-containing protein, site-specific integrase, hypothetical protein, hypothetical protein, RNA-binding domain-containing protein	SA-OH-C1, -C6	WP_046824851.1 (*Porphyromonas gingivalis* 95.1%), WP_042225841.1 (*Porphyromonas* sp. COT-290 OH860 98.8%), WP_036883452.1 (*Porphyromonas gingivicanis* 97.7%), WP_043898338.1 (*Po. gingivalis* 100%), WP_025843004.1 (*Por. gingivicanis* 98.3%)
Nuclease, UPF0114 family protein	SA-OH-C2	WP_028904643.1 (*Prevotella intermedia* 98.8%), WP_320788821.1 (*Prevotella* sp. 98.1%)

## Discussion

*O. hominis* was successfully isolated from six nasopharyngeal samples, creating, to the best of our knowledge, the first cultured isolate collection of the species from African samples. Colony morphology and growth characteristics were consistent with published reports from Australia; haemolysis was observed, a delayed phenomenon also described in some strains of *O. rhinotracheale* [16]; *β*-lactamase production and sensitivity to *β*-lactam antibiotics differed between strains. CfxA is a well characterized class A2 extended spectrum *β*-lactamase that hydrolyses penicillins and broad-spectrum cephalosporins. It is commonly present among oral bacteria of the class *Bacteroidia*, including *Prevotella*, *Porphyromonas* and *Bacteroides* [37,38]. In isolates SA-OH-C1, SA-OH-C6 and the previously published *O. hominis* genome OH-22803 from Thailand [2], the *cfxA* gene is present within transposon Tn4555 [39]. The transposon insertion sites differ between the South African and Thai examples but are in a similar region of the chromosome: within 200 kb of the terminus. The presence of *cfxA* appears to confer reduced susceptibility to *β*-lactam antibiotics, with the most profound effect observed against penicillin G.

By comparing infants who acquired *O. hominis* during the study period with those who were not colonized, we are able to identify differences in diversity and community membership that are associated with colonization by this species. Diversity increases following colonization with *O. hominis*, with the *Ornithobacterium* colonized individuals having significantly more diverse communities than non-carriers of the same age. The increased diversity is probably due to the establishment of not just one species, but several co-colonizers highlighted in the network analysis: *Suttonella*, *Rappaport*, *Helcococcus*, *Lwoffella*, *Moraxella* and an unclassified *Gracilibacteria*.

Three pathobiont ASVs were among the co-colonizers associated with *O. hominis* in Cluster 1A (Fig. 5): *M. lacunata*, *R. israeli* and *M. catarrhalis*. Although the latter is a low abundance ASV of a species that dominates the dataset, *M. lacunata* and *R. israeli* co-occur near exclusively with *O. hominis. M. lacunata* is a species carried in the upper respiratory tract and historically associated with opportunistic ocular infections [40], rarely causing soft tissue infection [41], bacteraemia [42] and endocarditis [43]. *R. israeli* is a recently described bacterium from the *Cardiobacteriaceae* family, thus far only observed in paediatric bacteraemia [44]. The consistent co-occurrence of *Suttonella*, *Rappaport*, *Helcococcus* and *Lwoffella* with *O. hominis* could indicate a biofilm community that aids *Ornithobacterium* survival in the nasopharynx and/or that benefits from *O. hominis* activity. Based on the appearance of *L. lincolnii* before colonization with *O. hominis*, and the relative rarity of these ASVs among non-carriers, carriage of this species may be a prerequisite for *O. hominis* acquisition.

Correlation Cluster 1B, consisting of ASVs that are not part of the *Ornithobacterium* subcluster, includes protective or health-associated species of *Dolosigranulum* and *Corynebacterium* [14] known to inhibit pneumococcal/staphylococcal growth. Notably, *O. hominis* is inversely correlated with corynebacteria in this dataset, particularly *Corynebacterium accolens*.

As a persistent colonizer of the upper respiratory tract, *O. hominis* may act as a reservoir of antibiotic resistance genes or virulence factors. Functional analysis of the genomes supports this, for example, in the over-representation of ‘replication and repair’ genes originating from mobile elements. We assessed the potential contribution of genes from other species and identified regions relating to antibiotic resistance, adhesion and toxin production that may have been acquired from other species through mobile elements or recombination.

Evidence of inter-species sharing of antimicrobial resistance genes is provided by the well-studied transposon Tn4555, a 10 kb transposon carrying the *cfxA β*-lactamase gene, found in SA-OH-C1 and -C6. Although this transposon is widespread, the nucleotide sequence present in *O. hominis* is >99.9% identical to that observed in *Prevotella scopos*, *Prevotella denticola*, *Prevotella melaninogenica* and *Prevotella distasonis* genomes, all of which are oral species of *Prevotella*.

Adhesins are another way in which bacteria can adapt to the host environment. All *O. hominis* genomes to date include a variable set of at least 20 FSD genes, many of which also contain fibronectin-binding domains and have an associated putative phase variation mechanism. Large complements of FSD genes are known in only a few other bacteria, such as *F. succinogenes* (a rumen commensal), from which they are named; *Chryseobacterium nematophagum* (a pathogen of nematodes); and *Elizabethkingia anophelis* (a mosquito gut commensal). A block of three exogenous genes in SA-OH-C1 and -C6 consists of a clostripain-like protease, a putative preprotein and an FSD gene similar to those in several *Prevotella* and *Alloprevotella* species, altogether sharing >99% nucleotide identity with the oral *Prevotella heparinolytica* strain F0111.

Finally, RHS toxin genes encode a large protein apparatus enclosing a C-terminal toxin that is delivered to a prokaryotic or eukaryotic target cell by a type VI secretion system [45]. The toxin tips are variable and change via a displacement mechanism, generating an array of pseudogene tips and immunity genes [46]. All six South African *O. hominis* isolates carried an RHS toxin tip and immunity gene within their arrays, which has also been observed in a group of *Bergeyella cardium* isolates [47] but has not been observed in *O. hominis* elsewhere. The *B. cardium* genomes have a similarly structured RHS array, although the primary RHS protein is of a different sequence to that of *O. hominis*, suggesting that toxin tips can be shared independent of the RHS context.

Interestingly, all of these matches were to oral species of bacteria rather than nasal colonizers. Recombination between nasal and oral bacteria has been described before, for example, driving the mosaic penicillin-binding protein diversity of *S. pneumoniae* and thus its acquisition of *β*-lactam resistance [48]. Although the analysis is based on few successfully cultured isolates of *O. hominis*, it provides preliminary evidence that the species may acquire genetic material from the microbiome; further work is required to demonstrate that this constitutes a functional reservoir of virulence factors and to identify specific instances of acquisition within the DCHS cohort.

A key virulence factor of Gram-negative bacteria is LPS, which triggers an inflammatory response from the host immune system [49]. As LPS is a highly immunogenic surface molecule, its biosynthetic gene clusters are common sites of recombination in bacteria [50,52]. The South African genomes are more similar to one another than to those from elsewhere (Fig. 1), but the close relationship between LPS biosynthetic gene clusters from different countries infers that these have been exchanged through recombination. The LPS operon appears to have been disrupted by an insertion event in three South African genomes, similar to a Cambodian example (Fig. 3). The putative transposon is at the same location in different LPS gene clusters, yielding the same 135 bp insertion site duplication. This may be due to independent events at a conserved target site or a historic insertion that was passed on through recombination at the LPS locus. There is no evidence for a dynamic or variable presence of this mobile element in the raw long-read sequence data from plate sweeps of multiple colonies, so it may no longer be functional. It is not known what the impact of an insertion on LPS biosynthesis may be.

## Conclusion

This study presents the first cultured isolate collection of *O. hominis* from African samples and represents a step forward in our understanding of *O. hominis* biology and its potential impact on human health. Genomic characterization reveals insights into potential antibiotic resistance mechanisms and demonstrates a potential role of *O. hominis* as a reservoir for virulence genes. Furthermore, network analysis reveals co-colonization patterns, suggesting *O. hominis* has an impact on nasopharyngeal microbial community structure. Despite the constraints of sample size and the wider limitation of gene annotation quality in the *Weeksellaceae*, it provides a foundation for future studies to explore the direct clinical relevance of *O. hominis* and its interactions within the nasopharyngeal microbiome.

## Further work

The findings of this project provide a basis for further work. Foremost, the clinical significance of *O. hominis* should be investigated, including relevant virulence factors such as toxin production and LPS. To address the difficulty of isolating *O. hominis* from primary material, optimization of culture strategies is also a priority. The presumptive cause of reduced *β*-lactam antibiotic susceptibility, CfxA, should be validated, as well as identification of the cause of apparent co-trimoxazole resistance. The relationships that are inferred through network analysis should be characterized to confirm whether they represent a mutualistic reliance, niche overlap or promotion of bystander growth; in doing so, it is also necessary to describe the novel species of *Helcococcus*, the genome of which is described in Table S2.

## Limitations

The main limitation of this study is the small number of isolates and genomes, which reduces the ability to infer population structure. More isolates and genomes are required to address this issue. Another is the large fraction of hypothetical genes (on average 12% of genes) for which we cannot infer a function. This is perhaps a reflection of poor characterization of novel genes across the *Weeksellaceae* family (for example, *Weeksella virosa* with 16% hypothetical genes, *Chryseobacterium indologenes* with 20% or *O. rhinotracheale* with 25%) compared to well-studied species such as *Escherichia coli* for which fewer than 5% genes are uncharacterized. The lack of annotation data limits our ability to contextualize gene functions and constrains our understanding of *O. hominis* biology. Functional validation of genes is required to address this lack of data.

## Supplementary material

10.1099/mgen.0.001635 Uncited Supplementary Material 1.

## Abbreviations

ANI: average nucleotide identity
ASV: actual sequence variant
CARD: Comprehensive Antibiotic Resistance Database
COGs: clusters of orthologous groups
DCHS: Drakenstein Child Health Study
FSD: *Fibrobacter succinogenes* domain
HCLD: HIV-associated chronic lung disease
IS: insertion sequence
LPS: lipopolysaccharide
RGI: resistance gene identifier
